# The longitudinal integrated database for health insurance and labour market studies (LISA) and its use in medical research

**DOI:** 10.1007/s10654-019-00511-8

**Published:** 2019-03-30

**Authors:** Jonas F. Ludvigsson, Pia Svedberg, Ola Olén, Gustaf Bruze, Martin Neovius

**Affiliations:** 1 Department of Medical Epidemiology and Biostatistics, Karolinska Institutet, Stockholm, Sweden; 2 Department of Paediatrics, Örebro University Hospital, Örebro, Sweden; 3 Division of Epidemiology and Public Health, School of Medicine, University of Nottingham, Clinical Sciences Building 2, City Hospital, Nottingham, UK; 4 Department of Medicine, Columbia University College of Physicians and Surgeons, New York, NY USA; 5 Clinical Epidemiology Unit, Department of Medicine Stockholm, Karolinska Institutet, Stockholm, Sweden; 6 Division of Insurance Medicine, Department of Clinical Neuroscience, Karolinska Institutet, Stockholm, Sweden

**Keywords:** Education, Income, Labour market, Occupation, Social support, Sweden

## Abstract

Education, income, and occupation are factors known to affect health and disease. In this review we describe the Swedish Longitudinal Integrated Database for Health Insurance and Labour Market Studies (LISA, Longitudinell Integrationsdatabas för Sjukförsäkrings- och Arbetsmarknadsstudier). LISA covers the adult Swedish population aged ≥ 16 years registered on December 31 each year since 1990 (since 2010 individuals aged ≥ 15 years). The database was launched in response to rising levels of sick leave in the country. Participation in Swedish government-administered registers such as LISA is compulsory, and hence selection bias is minimized. The LISA database allows researchers to identify individuals who do not work because of injury, disease, or rehabilitation. It contains data on sick leave and disability pension based on calendar year. LISA also includes information on unemployment benefits, disposable income, social welfare payments, civil status, and migration. During 2000–2017, an average of 97,000 individuals immigrated to Sweden each year. This corresponds to about 1% of the Swedish population (10 million people in 2017). Data on occupation have a completeness of 95%. Income data consist primarily of income from employment, capital, and allowances, including parental allowance. In Sweden, work force participation is around 80% (2017: overall: 79.1%; men 80.3% and women 77.9%). Education data are available in > 98% of all individuals aged 25–64 years, with an estimated accuracy for highest attained level of education of 85%. Some information on civil status, income, education, and employment before 1990 can be obtained through the Population and Housing Census data (FoB, Folk- och bostadsräkningen).

## Introduction

There has been a growing awareness of the importance of education, income, and occupation for the health of individuals and their families [1–32]. The purpose of the Swedish Longitudinal Integrated Database for Health Insurance and Labour Market Studies (LISA, Swedish: Longitudinell Integrationsdatabas för Sjukförsäkrings- och Arbetsmarknadsstudier) is to serve as the basis for statistics in health and labour market research. Through its annual data, researchers can follow individuals’ education, income, occupation, and employment by calendar year. These data are used to examine individuals’ life situation in relation to the labour market, working life, and ill-health. Labour market mobility, regional labour market conditions, longitudinal changes of the labour market, and the effect of social security, including sick leave benefit reforms on the labour market, can be studied. LISA is also used to track the training and skills of Swedish workers over time using their highest attained education.

This review aims to present and explain the contents of LISA. A second aim is to present medical studies in which components of LISA have been used. Today, most medical researchers use LISA to retrieve information on (a) education (as a covariate or matching variable), (b) sick leave and disability pension, and (c) unemployment.

### Relationship to population and housing census data (Folk- och bostadsräkning; FoB)

Every 5 years between 1960 and 1990, the government agency Statistics Sweden sent out a questionnaire to collect data on variables such as age, sex, civil status, country of birth, citizenship, most recent immigration, education, income, socioeconomic status, employment, and occupation from all Swedish adults aged ≥ 16 years. Swedish law stipulated that these surveys had to be completed. Consequently response rates were high. The results of these self-reported surveys make up the Population and Housing Census data. Although the last FoB survey coincided with the first year of the LISA database, only a small proportion of LISA data from 1990 originate from this last FoB survey (e.g., socioeconomic index, *SEI* 1990, and the Nordic classification of occupation, *NYK* 1990). Most data from LISA 1990 have other sources and are not self-reported. Nowadays, most of the variables in the FoB are collected automatically and found in the register RAMS (Labour statistics based on administrative sources), which is part of LISA.

## Contents of LISA

While the formal decision to initiate LISA was taken in 2003, Statistics Sweden compiled information starting in 1990. Thus, the register now contains annual data since 1990, using information from the Swedish Social Insurance Agency [Swedish: “Försäkringskassan”]) for sick leave and disability pension, unemployment data from the Swedish Public Employment Service (Swedish: “Arbetsförmedlingen”), and education data from the Education Register (Swedish: “Utbildningsregistret”). A list of all data sources for LISA can be found on the register’s web page [33]. Currently, the LISA database is updated with new annual data with a roughly 15-month delay. It contains some 500 variables on individuals, roughly 100 variables on firms and 30 variables on workplaces where Swedish adults work.

LISA is based on all individuals aged ≥ 16 years (since 2010, 15-year-olds are included as an adjustment to European Union regulations) who are registered in Sweden as of December 31 each year (and alive on that date) and thereby part of the Total Population Register (TPR) [34]. However, not all statistics in the annual LISA batch originate from December 31; for instance, LISA data from the RAMS database originate from November each year and data on retirement pension originate either from December 31 or the last payment in a specific year. For medical research purposes, these exceptions are rarely relevant.

Through linkage with the TPR [34], LISA also contains data on year and month of immigration and emigration and whether an individual has died during a specified 12-month cycle (after that, the individual does not occur in future editions of LISA). For instance an individual aged ≥ 15 years dying in February 2019 is included in the 2018 edition of LISA but not in the 2019 edition. Linkage to the TPR also ensures that household data are available, and through other linkages, LISA offers data on employment site and type of firm (“Appendix”).

Data in LISA are based on a number of sources, including the TPR, RAMS, the Education Register, Register of Income and Taxation, Occupation Register, 1990 Population and Housing Census (“FoB 1990”), Structural Business Statistics from Statistics Sweden, the Swedish Social Insurance Agency, and the Swedish Public Employment Service.

### Linking data

#### Individuals

Most linkages in LISA use the personal identity number (PIN) as the unique identifier [35]. The study population in the LISA register corresponds to all individuals aged ≥ 16 years in the TPR (since 2010, ≥ 15 years). Because of delayed reporting of deaths and emigration, the TPR has a small over-coverage (likely about 0.5%) [34]. Additional details on emigration/immigration, movements within Sweden, and students with transient accommodation can be found elsewhere [34].

#### Employer data

All firms in Sweden that hire workers for pay are required to report the identity and salary of the employees to the Swedish tax authorities. This information (Swedish: “kontrolluppgift”) is used by Statistics Sweden to construct variables describing the employers of employed individuals in LISA, but also the employees and the self-employed. Additional data on employers are listed in the “Appendix”.

### Demographic variables

The LISA database contains information on age (through birth date) and sex. Similar to most other national registers in Sweden, “1” denotes men and “2” women in the LISA database. For a discussion on changes of the PIN, change of sex, and change of age, we refer to our earlier paper on the PIN [35].

In LISA there is also information on county and place of residence as of December 31 each year. Up until 2014, there were data on county, city and parish (Swedish: Län, kommun och församling), but in 2015 parish was replaced by district. Since 1991, university students should be registered as living in their university city [34], but a large proportion of students who have moved from their home are still registered at their parents’ place of residence.

### Civil status

Civil status is coded as unmarried (OG), married (G), divorced/separated (S), widow(er) (Ä), registered partner (RP; judicial term for same-sex marriage), divorced partner (SP), and surviving partner (EP). Since May 2009, same-sex marriage is allowed in Sweden and after that date no person has been assigned the RP code. A person who has been an RP before May 2009 but never married, will remain RP but classified as EP after the death of the partner.

Since July 2014, it is illegal to marry in Sweden before age 18 years. However, a small number of individuals < 18 years and not Swedish citizens at the time have married abroad and are then coded as married. LISA also registers the number of years in the latest civil status (for unmarried individuals, the number of years equals age).

### Migration, country of birth, foreign background, and nationality

LISA includes data on the last year and month of *immigration*, as well as the country from which the individual has migrated (the standard annual version of LISA contains the latest immigration/emigration, but it is possible to obtain data on *all* migrations through the database). A special case concerns migration to the neighbouring Nordic countries. When a Swedish citizen moves to another Nordic country [34], the rules of the population registration in the host country apply. Accordingly, when another country (e.g., Norway) registers individuals as living in Norway (after more than a 6-month stay), they are automatically unregistered in Sweden.

**Fig. 1:**
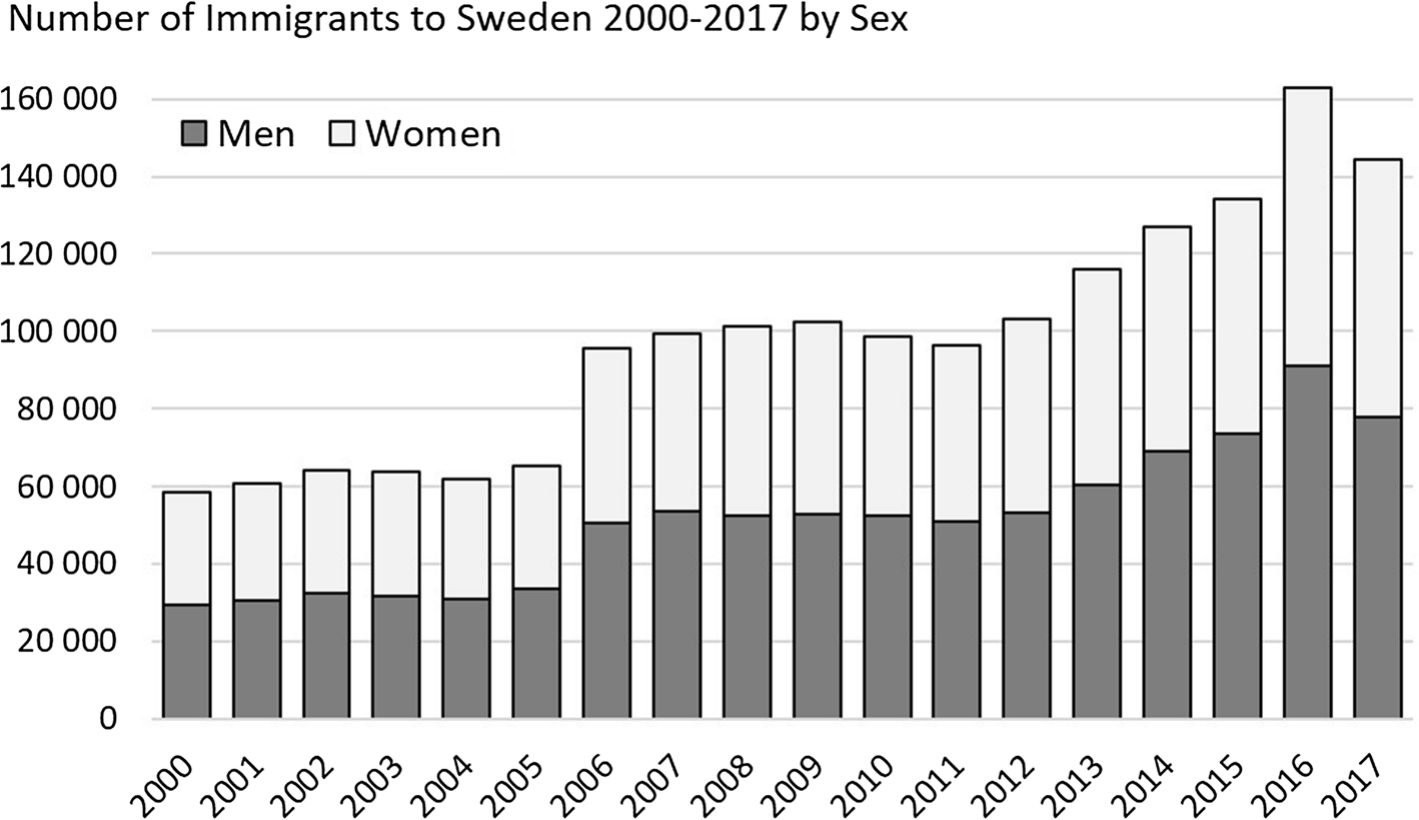
Number of immigrants to Sweden annually between 2000 and 2017

From 2000 to 2017, an average 97,000 individuals immigrated to Sweden annually, with the number increasing steadily each year from 58,659 in 2000 to 163,005 in 2016 (Fig. 1) and then 144,489 in 2017. Hence annual immigration corresponds to about 1% of Sweden’s population (currently about 10 million). Many immigrants originate from the Middle East (primarily Syria and Iraq), Northeast Africa (Somalia and Eritrea), and the Balkans. Also EU immigrants (the other Nordic countries, especially Finland; but also Poland and Germany) make up a substantial part of immigration to Sweden. In 2017, 15% of all immigrants came from Syria, with the second largest group of immigrants by country of birth being returning Swedes (9% in 2016).

Until 1947, all children born abroad were registered as *foreign*-*born*; however, since 1947, a child born abroad but to a mother registered as living in Sweden is recorded as born in Sweden. From 1998, also children born to a father registered in Sweden were recorded as born in Sweden. LISA uses four classifications for country of birth (EU15 [“Appendix”], EU25, EU27, EU28). Although LISA contains data on exact country of birth, Statistics Sweden rarely deliver data on that level of detail to medical researchers. See our previous paper [34] for a discussion of country of birth when a country has ceased to exist (e.g., the Soviet Union and Yugoslavia).

Parents living in Sweden at some point since 1947 when the PIN was introduced [35] are linked to an index individual [their child(ren)] in the LISA database. Since 1950, at least 99% of all individuals in LISA have data on the identity of the mother, but for the period 1932–1949, the proportion is lower. Only 4% of foreign-born who immigrated to Sweden at age ≥ 18 years have data on the identity of their mother. Data on paternal identity became near complete (≥ 99%) in 1967. Data on *parental country of birth* is very limited among individuals born before 1932 (see paper on the Multigeneration Register) [36].

Two additional variables that relate to foreign background are *UtlSvBakg* and *UtlSvBakgAlt*, where the latter is more detailed. These variables note whether a person was born inside or outside Sweden and whether the person has (0, 1, or 2) parents born in Sweden.

For a discussion of citizenship, see the “Appendix” and our earlier paper on the TPR [34].

### Education variables

Since 1990, LISA contains data on the highest attained education by calendar year through the Education Register (UREG) [37]. Data in the Education Register, based on data from more than 30 sources, are coded pursuant to the SUN2000 nomenclature (Swedish education nomenclature). These sources include compulsory school, universities, the military academy, agriculture schools, questionnaire data from immigrants, studies at foreign universities, folk high schools (these do not award academic degrees), employment agency databases, etc. An estimated 1.7% aged 25–64 years have missing data on education (mainly individuals born outside Sweden). Education data from immigrants are obtained from the Swedish Migration Authority, Government-sponsored Swedish language courses for immigrants (language training; Swedish: Svenska för invandrare: SFI), and the Swedish Public Employment Agency (Swedish: Arbetsförmedlingen). Since 1999, an annual questionnaire is sent out to all immigrants whose education data is missing and who have arrived that year. The response rate of the questionnaire is generally about 50%. As soon as an immigrant takes part in any educational activity in Sweden, that (new) level of education will override the older, self-reported data.

The quality of education data in the Education Register, and included in LISA, has been evaluated several times. A 2006 evaluation indicated that level and type of education were correct for 85% of individuals at the time. The validity is higher in individuals born in Sweden. Some 99% of individuals with a registered education ≥ 3 years of university studies have attained this level of education, but because higher education is not always recorded, the proportion of individuals with low education (as registered in LISA) is probably overestimated. Given the revision of the Education Register in 2000, data before and after that year should be compared cautiously (as a result of the revision, the general level of education rose substantially in Sweden in 2000). Before 2000, Statistics Sweden used an “old SUN classification”. The newer SUN2000 is adapted to the international nomenclature ISCED 97 (International Standard Classification of Education). In 2004, a new system was applied to obtain data on education. This increased the number of engineers, physicians, and economists in Sweden.

*Statistics Sweden* have access to education data since 1985, but only data from 1990 and onwards are included in LISA.

**Fig. 2:**
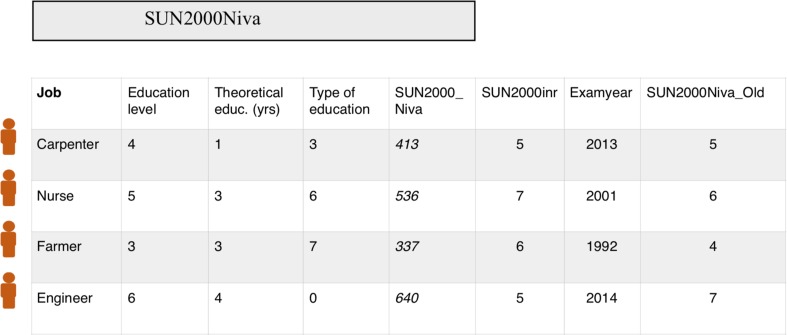
Levels of education. Theoretical education denotes years of education in the highest education category a person belongs to. Persons who have completed 3 years of upper secondary school (and no college/university education) will receive a code for “upper secondary” and the value “3” (3 years of theoretical education in their education category). Values in Fig. 2 may not represent all persons belonging to a certain career type (for instance, there are of course farmers with a theoretical education). The easiest way to retrieve data on education from LISA is to use the seven levels of SUN2000Niva_Old (1 = not completed compulsory education (< 9 years), 2 = completed compulsory education (9 years), 3 = upper secondary (2 years), 4 = upper secondary (3 years), 5 = college/university < 3 years, 6 = college/university ≥ 3 years, 7 = research education). In the newer SUN2000 the levels 3 + 4 from SUN2000Niva_Old have been merged (value “3”). This means that the new SUN2000NIVA ranges only has six levels

SUN2000Niva denotes the *level* of education, whereas SUN2000Inr (Inr, *inriktning*; Swedish abbreviation for specialization) denotes the *type* of education (if you become a nurse, an engineer, etc.). These two variables have replaced the Old SUN classification which did not distinguish between level and type of education. However, for practical reasons, the SUN2000Niva_Old is often used by researchers to retrieve data on education (see legend, Fig. 2). Even if SUN2000Niva_Old was exclusively used in 1990–1999, the new SUN2000Niva is recoded into SUN2000Niva_Old (similar to the back-coding of ICD8-10 to ICD7 in the Swedish Cancer Register [38]). Hence, SUN2000Niva_Old allows for longitudinal studies of education since 1990.

The current SUN2000Niva has three digit positions (Fig. 2). The first digit position ranges from 0 to 8 (from < 9 years of education to research level education), with 9 representing missing data. The second digit position represents the number of years of a theoretical education after compulsory school. Thus, for individuals with ≤ 9 years of schooling, the second digit is “0” (this is most often seen for older people or those born outside Sweden). A smaller number of people at level 6 (research education) have “0” in the second position because it is unknown whether they have finished their research education after 2 years (licentiate) or 4 years (Ph.D.). The third digit position represents the type of education credentials held by a person. The legend of Fig. 2 describes the translation between SUN2000Niva and SUN2000Niva_Old.

SUN2000Inr has four positions. The first two digits are similar to the ISCED 97 classification (for instance, “2” is education in the Arts while “7” refers to health care and social services). SUN2000 has been grouped into 97 groups of education (*SUN2000Grp*). There is also a variable for examination year; however, because not everyone has passed an examination, this variable (*ExamAr*) is often missing and thus should only be used prudently.

### Family-related demographic variables

Each family has a family identity number (*FamId*). This variable is equal to the PIN of the eldest family member when a maximum of two generations are living together (registered at the same address). Unmarried couples with joint children who cohabit have the same *FamId*. Unmarried couples without joint children who cohabit are not assigned the same *FamId*. In 2013, Statistics Sweden estimated that about 500,000 adults are cohabiting (Swedish: “sambo”) but with different *FamId* (they do not have joint children).

The LISA database does hence not contain data linking cohabiting unmarried couples without children. Such data have to be obtained separately from Statistics Sweden since 2011 through the Real Estate Register. Statistics Sweden then use an algorithm to indicate unrelated different-sex individuals with < 15 years of age difference living at the same address.

With the exception of cohabiting unmarried couples without children, all members of a family are assigned the same “family type”. Families are divided according to whether they consist of one or two adults, whether two spouses are married in a partnership or cohabiting, and whether they have children (no vs. yes [yes is divided into 3 groups: children < 18 years living at home vs. children ≥ 18 years living at home vs. children who have left home]). Additional information on number of children living at home is presented in the “Appendix”.

Except for single households (Swedish: ensamstående, code “400”), all individuals live in a relation (with a parent, child, partner, etc.). Statistics Sweden characterize all individuals according to their relationship within a household (e.g., a single father with several children ≥ 18 years is assigned the code “220”). Family relationships in LISA are coded under family status (Swedish: familjeställning: *FamStF*).

Families are also assigned consumption weights to facilitate a comparison of the living standards of families with different demographic compositions (“Appendix”).

### Occupation and employment variables

For occupational data, LISA depends on the Occupation Register. The main occupation is that with the highest taxable salary in November every year. The government agency is aware of at least two problems with the occupational data. First, not everyone pays an earnings tax, which reduces the available information on occupation, and second, some individuals may work but are not being paid until at a later time (or not even the same year). The latter happens especially to young people with summer work and to the self-employed.

From 2001, there are data on occupation ((*SSYK;* Swedish: Standard för svensk yrkesklassificering), completeness about 95%). Missing data include data from firms that have not reported to the authorities, certain union work, and project employees. More detailed data on occupation are available through *SSYK4* (one additional level of data), with a completeness of about 55–60% until 2003, and a completeness of 87–96% thereafter, except for the years 2014–2015 when a new nomenclature was introduced. Hence, *SSYK3* and *SSYK4* reflect the degree of details for occupational data (three or four digits, respectively) and should not be mixed up with *SSYK96* and *SSYK2012,* which categorizes occupations.

The earlier occupation classification in LISA, originating from the 1990 FoB, is based on self-reported occupation.

LISA also contains information on employment status (Swedish: “sysselsättningsstatus”), whose purpose is to describe whether an individual is working, with a definition of work that includes employment in a firm or self-employment. Statistics Sweden creates an estimated employment status for individuals in LISA based on several data sources. The main data source is reports on salaries paid to individual workers from firms to the Swedish tax authorities. Statistics Sweden combine these reports with information from surveys of the labour market to estimate the average number of hours per week an individual worked during a calendar year. Labour market surveys are used to obtain an annual salary cut-off that determines who is regarded as employed (the annual salary is calculated based on data from October–November each year). The cut-off is lower for women and very young and old people. For instance, in 2017, middle-aged men earning < circa 90,000 SEK per year were regarded as *not being employed*. However, self-employed people are considered employed, independently of their salary as registered in October–November provided that their company is recorded as active.

The definition of employment has otherwise changed several times (1990–2003, 2003–2011, 2011–), sometimes with overlap. The changes include the criteria for employment status among retirees and for women giving birth in a specific year (both corrections increased the absolute number of employed in Sweden). Changing rules for self-employed in 2004 resulted in the number of people in employment increasing in that year. Until 2001, LISA recorded employment in people aged ≥ 16 years. In 2002–2010 employment was registered in individuals aged 16–84 years. Since 2011, employment is registered for people aged 15–74 years to be consistent with the Swedish Labour Force Surveys (Swedish: “arbetskraftsundersökning, *AKU*”).

### Income and allowances

Income variables are listed as multiples of 100 SEK. For instance, a value of 50 is equal to 5000 SEK (roughly €500).

#### Earnings

LISA contains data on individual gross earnings (Swedish: “löneinkomst”, *LoneInk*) based on reports of salaries paid to individual workers from firms to the Swedish tax authorities and payments from firms to the self-employed who pay social security taxes. For the average working-age individual, earnings are the primary source of income.

#### Business income

LISA also contains several variables related to business income (Swedish: “inkomst av näringsverksamhet”). A business activity has to be independent, sustained, and profit maximizing if the income from the activity is to be classified as business income. Examples of income in this category are income from farms and other small businesses. The definition of the business income variables changed in 1991 and 2004.

#### Allowances to parents

Parental allowance (Swedish: “föräldrapenning”, *ForPeng*) is available to parents from the birth of a child (or date of adoption). In the first year both parents can request parental allowance for the same days; thereafter, the parenting payment is only available to one parent (either the mother or the father) at a time. Over the years, the maximum duration of the parental allowance has changed (currently 480 days). It can be requested up to the child’s 8th birthday or until the child has finished the first year in school. A portion of the parental leave is reserved for “the other parent”, i.e. a mother or a father of a child cannot use all 480 days (unless the child has no second parent). The minimum amount of days reserved to the *other parent* goes under the colloquial name “daddy months” (Swedish: “pappamånader”) and is currently 90 days. Under special circumstances (e.g., when a child needs inpatient care), parental allowance can be replaced by temporary parental allowance.

The Swedish Social Insurance Agency supports parents on leave from work in connection with the care of a sick child (temporary parental allowance, *ForVAB*). This payment is also available to a parent who must be away from work because of the child’s visit to dental, health, or psychiatric care, or needs to care for the child because the usual carer (often the other parent) needs to accompany another child to a health care facility (or similar). The temporary parental allowance is restricted to 60 days per child and year though it can be prolonged under special circumstances.

The father (or “the second mother” if the newborn/adopted child has two mothers) has the right to a temporary 10-day leave at the child’s birth or adoption. This short-term parental allowance is often referred to as “daddy days” (Swedish: “pappadagar”).

Until 2018, a parent to a child < 19 years with a handicap, intellectual disability, or severe somatic/psychiatric disease requiring special care or supervision for ≥ 6 months could claim care allowance (Swedish: “Vårdbidrag”, *VardBidr*). This allowance was re-evaluated every 2 years by the Swedish Social Insurance Agency. Municipal care allowance (Swedish: “kommunalt vårdbidrag”) was distinctly different from care allowance and was not paid in response to child illness, but a local low-level compensation offered in some Swedish cities (not all) to parents who stay at home with children aged < 3 years instead of using available municipal day care. Neither the Care allowance nor the Municipal care allowance is available anymore.

Related to parental allowances is the close relative (e.g., for mother, father, sister, brother, wife, husband) allowance (Swedish: “närståendepenning”, *NarPeng*) initiated in 1989. An individual has the right to take leave from work for up to 100 days to care for a close relative if both the carer and the sick person are registered with the social insurance system.

#### Sick leave

Several of the sickness allowances (but also the parental allowance) are based on a “sickness benefit-based income” (Swedish: “sjukpenninggrundande inkomst-*SGI*”), which is currently 97% of a person’s individual annual earnings. Benefits typically correspond to 80% of this amount (0.80 * 0.97) but are capped at a lower level for high-income earners (capped at 7.5 basic amounts annually for sick leave compensation). The basic amount (Swedish: “basbelopp”) has changed over the years, increasing about 50% (from approximately 30,000 SEK in 1990 to 45,500 SEK in 2018, “Appendix”: Fig. 6).

**Fig. 3:**
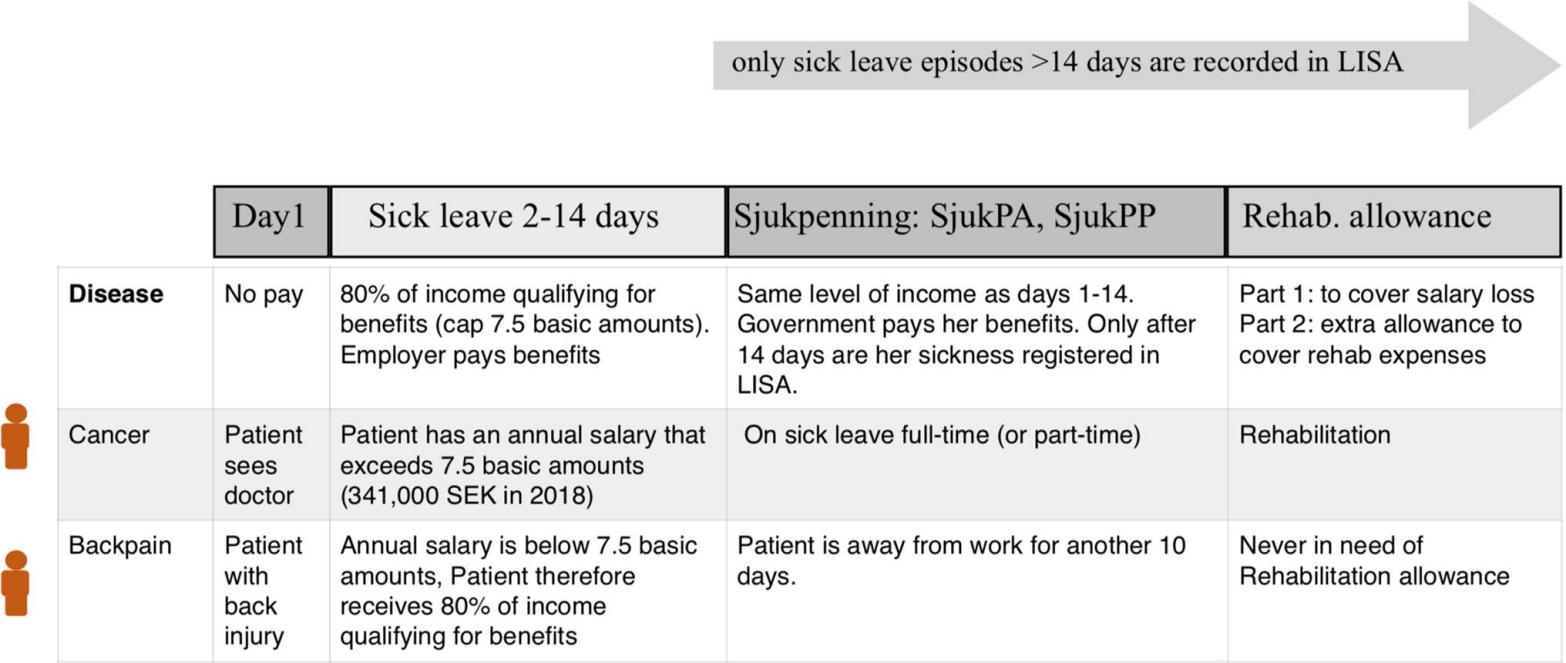
Sickness benefits for people aged 15–64 years. The LISA database only contains data on sick leave after the first 14 days. LISA data on sick leave originate from income data reported from the Swedish Social Insurance Agency (and from this it can be deduced that an individual is on sick leave). In 1990–1993, the sickness benefit after the first 14 days was registered by the variable SjukPA and since 1990 also as SjukPP. LISA contains data on days of sick leave as well as the actual income. Number of days always refers to the same year, but income for sickness in December will be paid in January the next year. Number of days is recorded by the variables SjukP_bdag/SjukP_ndag (and for rehabilitation allowance, Rehab_bdag/Rehab_ndag). Bdag represents the gross number of days away from work due to sickness, whereas Ndag is the net number of days (“full sickness days”; e.g., an individual is on sick leave all afternoons (50%) for 14 days. He or she will then have 14 SjukP_bdag but only 7 SjukP_ndag). In the future, Statistics Sweden plan to retrieve data on a monthly basis (rather than annually), which will facilitate studies on the relationship between timing of disease and prior/later work absence. The qualifying day was removed in Sweden in 2019

During the first 14 days of sick leave, the employers are responsible for sick pay. Because this pay cannot be differentiated from ordinary salary, Statistics Sweden cannot identify sick leave ≤ 14 days. If a sick leave episode is ≤ 14 days, the episode does not enter into the sick leave statistics in LISA. Previously, the first qualifying day had no cash benefits (Swedish: “karensdag”). On Jan 1, 2019 the government removed the qualifying day as some employees were affected more than others (especially people working evenings and weekends), and instead introduced a qualifying deduction. After the first 14 days, the individual can apply for sick leave benefit (Swedish: “sjukpenning”; *SjukPA* and *SjukPP*) (Fig. 3). For repeated sick leave episodes with a short interval in between and for patients with a chronic disease, the responsibility of the employer to compensate the first 14 days can be waived. The self-employed can choose to have 2 qualifying days or more without cash benefits (self-employed who are sick ARE paid by the Swedish Social Insurance Agency).

Sick leave benefits are paid only to individuals with an annual salary of at least 10,000 SEK (roughly €1000) and when the disease or injury compels the individuals to be away from work for ≥ 25% of the time. Other related allowances are pregnancy allowance (Swedish: “havandeskapspenning”) and contagious disease allowance (Swedish: “smittbärarpenning”). Pregnancy allowance is paid for ≤ 50 days, ending no later than 11 days before expected delivery. It targets women with physically exhausting work. However, because the definition is widely interpreted, most pregnant women who apply are likely to be granted this allowance. Contagious disease allowance is paid to someone who, because of the risk of disease transmission, is ordered by the authorities not to attend work. The level of benefits has changed with time, often reflecting the labour market and unemployment levels.

Rehabilitation allowance (Swedish: “rehabiliteringsersättning”, *RehabErs*) has been available since 1992 and consists of two parts: a direct payment to the individual to cover salary loss and an extra allowance to compensate for increased expenditures owing to the rehabilitation per se. Individuals undergoing rehabilitation or suffering from disease can also receive a separate housing allowance (registered in LISA).

#### Disability pension

Disability pension (Swedish: “förtidspension”) was available until 2002 for individuals aged 16–64 years with permanent work incapacity (at least 25% reduced work capacity was required). In these years *temporary* (or short-term) disability was compensated through a sickness allowance (Swedish: “sjukbidrag”).

Since 2003, individuals aged 30–64 years with long-term work disability are entitled to sickness compensation (Swedish: “sjukersättning”, *SjukErs*) [39] and younger individuals (19–29 years) are eligible for activity benefit (Swedish: “aktivitetsersättning”). Both types of benefit correspond to disability pension. As before, both of these allowances require at least 25% reduced work capacity and are part of the social insurance system. Individuals on disability pension until 2002 automatically received sickness compensation from 2003. While sickness compensation can be permanent, activity benefit is always temporary but refers to work disability expected to last at least 1 year. Since 2009, individuals with sickness compensation or activity benefit are allowed to work a small amount but retain the full compensation (for instance ≤ 5 h per week with political or voluntary work paid < 12.5% of the normal weekly salary of an individual is accepted).

#### Unemployment

Certain LISA data on unemployment originate from the Swedish Public Employment Service (Swedish: “Arbetsförmedlingen”). These data include days of unemployment benefits and days in an active labour market policy measure (Swedish:”aktiv arbetsmarknadsåtgärd”).

**Fig. 4:**
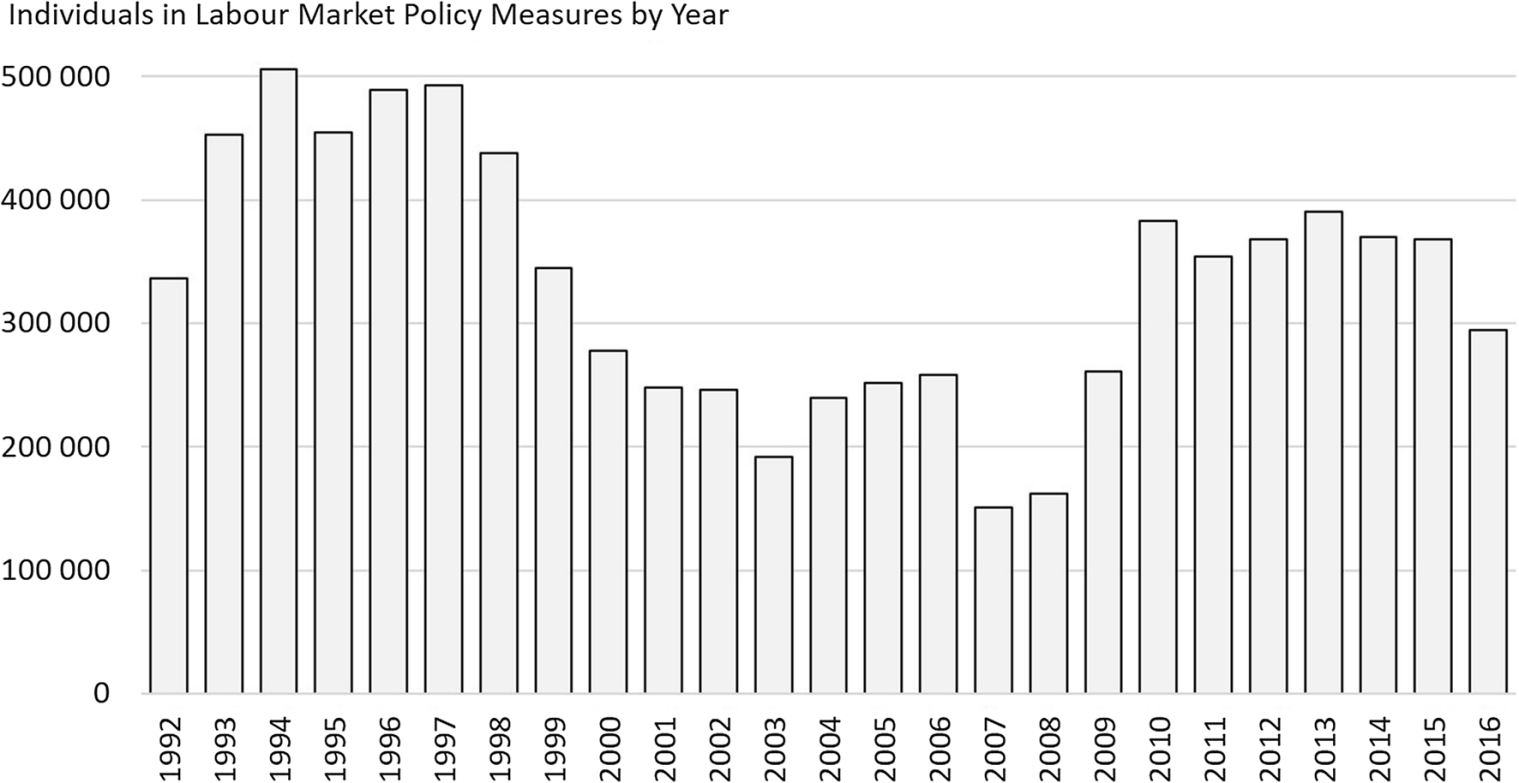
Number of individuals in labour market policy measures in Sweden by year

Politically motivated actions serving to support/activate individuals outside the regular labour market are known as “labour market policy measures”. The number of participants in such policy measures has varied between 150,000 and 500,000 in 1992–2016 (Fig. 4).

Unemployed individuals have the right to unemployment benefits until the age of 65 years (this requires that the individual fulfils certain criteria and is ready to take a suitable job offered by the Swedish Public Employment Service). Both amount (employment benefits in multiples of 100 SEK, arbetslöshetskassa or *AKassa* for short) and the number of days (*ALosDag*) receiving such benefits are recorded. Benefits can be paid for full- or part-time employment (but the degree of time loss cannot be differentiated). Currently, the maximum length of unemployment benefits is 300 days (special rules apply to parents with minors). A special kind of unemployment benefit was the *KAS* allowances paid out during the 1990s (Swedish: “Kontant arbetsmarknadsstöd”).

LISA also records education allowances that serve to improve unemployed people’s chances of gaining employment (Swedish: “aktivitetsstöd/utbildningsidrag”, *UtbBidr*). These allowances are usually granted to younger people. Since 2011, immigrants may be entitled to allowances that aim to help them establish themselves on the labour market (*EtabErs*).

#### Other income

The LISA register also records data on income from capital (Swedish: “kapitalinkomst”) and data on pensions from the National Retirement Pension Scheme. The retirement/old age pension includes several parts and are summed up in two variables: *SumAld* (1990–2002) and *SumAldP03* (2003 and onwards). To this money is added an income retirement pension to retirees born in 1938 and later (*InkPens*). The retirement pension is based on the number of previous working years, the salary development among the employed, and the age at which an individual retires.

During the years 1990–2004, individuals aged 60–64 years were allowed to gradually decrease their working hours (from 40 to 17–35) and receive a part-time retirement pension (Swedish: “Delpension”, *DelPens*). Certain conditions applied.

Another form of pension is the occupational pension (Swedish: “Avtalspension/Tjänstepension”). This pension is paid by an individual’s employer and depends on the salary and number of years at work. The total amount of occupational pension is recorded as the variable *SumTjP*.

#### Social welfare/general assistance

In compliance with the Swedish law for social services (Swedish: “Socialtjänstlagen”), the social security system must provide every resident the minimum resources considered necessary for a decent standard of life (Swedish: “försörjning och livsföring i övrigt”). This provision should cover food, rent, and other basic expenditures. The law applies to individuals residing in a Swedish municipality provided that they try to support themselves (e.g., apply for work when unemployed). Groups that often require social support include families with low incomes, the unemployed where unemployment support is insufficient, people in conflict at work, and people who are unable to work outside the home because of small children. Refugees and immigrants are also entitled to social welfare. Regrettably, LISA records for social support are incomplete because many local authorities have failed to report data to Statistics Sweden.

Family social welfare (Swedish: “socialbidrag för familj”, *SocBidrFam*; in multiples of 100 SEK per year) is the most common social welfare. The majority of recipients are single households or single parents with children. Part of these benefits is composed of housing support (variables usually begin with *Bost*).

Disabled individuals aged 19–65 years can request disability allowance (*HKapErs*). Disability allowance was available also to individuals aged 16–18 years until 2003 but these individuals now receive another type of benefit. Conditions for this allowance are listed in the “Appendix”.

Children with separated parents may be entitled to maintenance support (alimony; Swedish: underhållsstöd/bidragsförskott, *BidrFor*).

#### Disposable income

Disposable income is defined as total income received (measured from registers, including allowances) minus taxes paid (*DispInk* or *DispInk04*). A family also has a joint disposable income (*DispInkFam04* since 2004 and *DispInkFam* before 2004).

The disposable income is also presented “per consumption unit” (Swedish abbreviation KE), and then registered as *DispInkKE04 (before 2004: DispInkKE,* and with slightly different weights to calculate disposable income). In a family of two adults (consumption weight: adult 1 is 1.0 and adult 2 is 0.51) and two children (first child is 0.52 and second 0.42) the total consumption weight is 2.45.

**Fig. 5:**
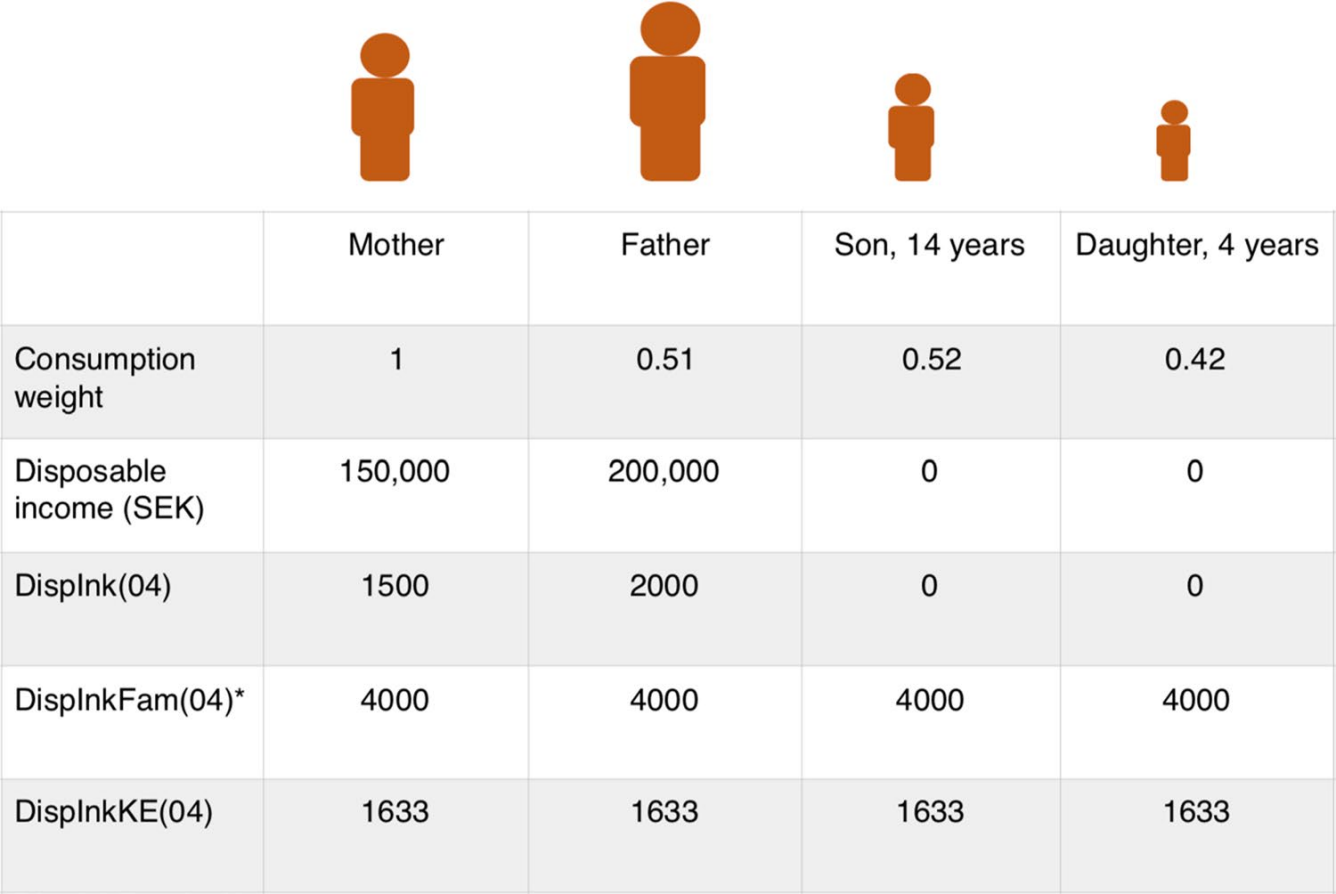
Disposable income. After taxes and allowances, the mother earns 150,000 SEK and the father 200,000 SEK for the year. In this family the son earns money (50,000 SEK, but he is not included in LISA because he is < 15 years old). The total disposable income of the family, however, is 400,000 SEK (represented by the value of DispInkFam(04) assigned to all individuals in the family). When divided by the consumption weight of this family (2.45), each family member is assigned the value 1633 (representing 163,300 SEK). In LISA, all statistics on income represent multiples of 100 SEK

**Fig. 6:**
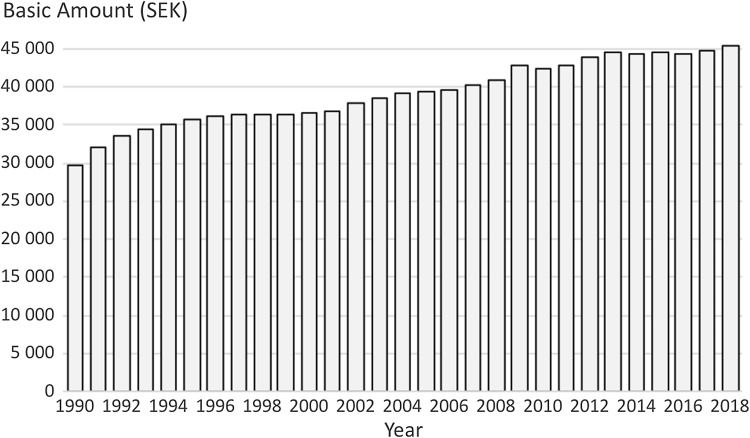
Basic amount (Swedish: “basbelopp”)

Figure 5 illustrates the relationship between individual disposable income (total income minus taxes), the disposable income of the family, and the disposable income per consumption unit. All income variables in LISA are in multiples of 100 SEK. Individuals in a family can have different *DispInk/04* but always have the same *DispInkFam/04* and *DispInkKE*/*04*.

### Socioeconomic indices

Indices on socioeconomic status can be retrieved through LISA from three periods. The socioeconomic index (SEI) coding is based on the *FoB* and reflects self-reported occupation in adults. SEI is only available for year 1990, and 8–9% of participants have missing data. LISA does not contain any data on socioeconomic index 1991–2000, but there are data on education and income. From 2001 to 2013, socioeconomic data are available through the occupational classification system (YSEG (Yrkesbaserad SocioEkonomisk Grupp), based on the variable *SSYK96*) and from 2014 to the present through the European Socioeconomic Classification System [ESeG, based on the variable *SSYK2012* (in itself based on < ISCO-08)].

SEI, YSEG and ESeG are constructed according to different principles and cannot be translated between each other.

YSEG was created based on the European Socioeconomic Classification (ESEC). ESEC, however, was never fully implemented in Europe because the work on the ESeG had already started.

The ESeG comprises nine groups (1–9, see “Appendix”) and 42 subgroups. Groups 1–7 refer to the active population (where labour status can be either employed or not employed). Individuals who are not employed are classified in a similar manner but based on their previous occupation. While ESeG is described as a socioeconomic classification it is really based on occupation.

### Medical use of LISA

Many researchers use LISA for medical research [1–32], when retrieving data on education [40–49], income [40, 50–52] (including family income [53, 54]), civil status [40, 42], unemployment [50, 52, 55–57], and work disability benefits [1, 50, 58] in association with many diseases and disorders. LISA data can be used to compare responders and non-responders in questionnaire studies. In one study, postal questionnaire non-responders had lower education, lower income, and were more likely to have been born outside Sweden than responders [59, 60]. Education and income can be used both as exposures and outcomes, but are most often used as covariates in statistical models [61, 62].

LISA contains data on health and neighbourhood deprivation [63–65] and allows researchers to explore the potential impact of socioeconomic status for disease development, but also the reverse (i.e. if disease is associated with moving to more deprived areas) [66].

One Swedish study described how disposable income has changed and analysed whether development of disposable income differed between working-age people with multiple sclerosis and a reference group [67]. Gyllensten et al. [4], investigating cost of illness of working-aged individuals in Sweden with multiple sclerosis, found that costs resulting from lost productivity contributed to approximately 75% of the estimated overall cost of illness in working-age patients with multiple sclerosis.

The influence of family structure on the probability of being granted disability pension has been investigated in young women and the highest risks were seen for single working mothers [28].

The incidence of type 1 diabetes is increasing in children and youth, suggesting that environmental factors are influencing those with a genetic predisposition [68]. Linking the Swedish Child Diabetes Register and socioeconomic data, researchers have studied the association between education, employment, and earnings [69–72]. Adult type 1 diabetes is associated with employment and salary development [73].

## Discussion

The LISA database was launched in response to an increase in sick leave and was initially seen as a tool to investigate changes in the labour market. It contains annual data since 1990. The extensive data on education, occupation, income, and various allowances (including social welfare), as well as data on work absence benefits due to injury, disease, or rehabilitation entail that the database has quickly developed into an important source of information for medical researchers. Data from the LISA database have been used not only as exposures and outcomes but also as covariates to reduce potential confounding and describe study populations.

As with all databases, LISA has strengths and weaknesses. Although it started in 2003, backtracking of information means that for many variables there are data over a period of 20–30 years, and sometimes even longer. Because the database is nationwide, it includes all individuals aged ≥ 15 years (≥ 16 years between 1990 and 2009) and living in Sweden. Importantly, participation in the Swedish government-administered registers is compulsory. Compulsory participation eliminates any selection bias, and through the LISA database, researchers have access to data even on social welfare benefits. Through linkage with the TPR, it is possible to calculate the disposable income of an individual, disposable family income (aggregated), and weighted income as per consumption unit.

The LISA database has some limitations. It is an administrative database, and as such, its primary purpose has not been to supply researchers with medical data. Medical researchers may desire more details on medical conditions that are not available in LISA. Still, linkage to the national health care registers can compensate for much of this information gap. More importantly, the database reflects the political landscape, where different forms of allowances, rules, and regulations have changed, often many times over the years, sometimes offering a fragmented view of certain aspects of income and allowances. For sick leave and disability pension, LISA provides data aggregated by calendar year, which are often detailed enough when used as indicators of frailty. However, investigating the impact of pharmaceutical or surgical interventions or acute health events, researchers may need day-level rather than year-level data [52, 74]. These data can be retrieved from the database MiDAS kept by the Swedish Social Insurance Agency [Swedish*: MikroData för Analys av Socialförsäkringen* (www.forsakringskassan.se)]. Moreover, LISA does not contain data on the underlying reason for sick leave (a person may be absent from work for different reasons in the same year). Despite its shortcomings, the LISA database has proven to be useful in medical research.

In conclusion, the LISA database is a useful tool for medical researchers to examine the relationship between labour market participation and health, or to adjust for factors such as education, income, and occupation to understand health and disease development.

## Appendix

### Employer data

All firms in Sweden hiring workers for pay are required to report the identity of the employees and their salary to the Swedish tax authorities. The information (Swedish: “kontrolluppgift”) is used by Statistics Sweden to construct variables describing the employers of employed individuals in LISA.

Formally, the requirement to report salaries is imposed on a firm (a legal entity that is required by law to file a tax return). A single firm may also be divided into different establishments (a geographically separate workplace unit that is typically engaged in the production of a smaller set of goods or services). A firm may thus consist of one or several establishments. Statistics Sweden divide firms into establishments based on information in the mandatory reports from firms to the tax authorities, the geographic location of the workplace, and other relevant parameters. LISA contains variables describing the employer of an individual both at the firm and establishment level. Examples of employer variables in LISA are the sector (e.g., the private sector), the industry (e.g., mining), and the municipality where an individual is working.

If an individual works for several firms, LISA includes information on up to three employers, with the main employer defined as the entity that pays the largest fraction of the salary to the individual.

### Disabled individuals: conditions for allowance

Disabled individuals aged 19–65 years can request disability allowance (*HKapErs*; until 2003, disability allowance was available to individuals aged 16–65 years). Conditions for this allowance include that the individual either a) must have a handicap that affects daily living, b) needs external support to be able to work, c) has a handicap that involves substantial extra costs, *or* d) is blind, deaf or has severe hearing impairment.

### Country classifications: EU15 (FodGrEg)

The LISA database uses the same country categorization for citizenship as for country of birth. 0 = Sweden; 1 = Nordic countries (other than Sweden, i.e. Denmark, Finland, Norway, Iceland); 2 = EU15 (except Denmark, Finland, and Sweden); 3 = Europe (except EU15 and the Nordic countries); 4 = Africa; 5 = North America; 6 = South America; 7 = Asia; 8 = Oceania; 9 = Soviet Union (when existing); 11 = Unknown (nobody is assigned “10”). Russia has the value “3” while other countries in the former Soviet Union will be assigned a value according to their geographic location. Statistics Sweden also uses other country classifications.

### Citizenship

Individuals with dual citizenship (one of which is Swedish) will only have their Swedish citizenship recorded in LISA (see also our paper on the Total Population Register). Before July 1, 1979, a child to a non-Swedish father and a Swedish mother was automatically assigned the nationality of the father, but since then children with at least one Swedish parent are assigned Swedish citizenship. For individuals born in Sweden, the date of birth is equal to date of Swedish citizenship.

### YSEG groups (occupational classification, Swedish: Yrkesbaserad SocioEkonomisk Grupp) (10 groups)

1 = Professionals and managers, 2 = Qualified civil servants, 3 = Other civil servants, 4 = Small entrepreneurs, 5 = Farmers, 6 = Work leaders and technicians, 7 = Business, service and welfare/care, 8 = Skilled workers, 9 Other workers, 10 = Not working.

### ESEG groups (European Socioeconomic Classification System, but actually an occupational classification) (9 groups)

1 = Managers, 2 = Professionals, 3 = Technicians and associate professional employees, 4 = Small entrepreneurs, 5 = Clerks and skilled service employees, 6 = Skilled industrial employees, 7 = Lower status employees, 8 = Retired persons (≥ 65 years), 9 = Other persons < 65 years outside the labour force (e.g., students),

### Number of children living at home

The number of children living at home is further classified as number of children aged 0–3, 4–6, 7–10, 11–15, 16–17, and ≥ 18 years (“Barn18plus”). In later years two new categories have been created representing children aged 18–19 years (“Barn18_19”) and ≥ 20 years (“Barn20plus”).

### Consumption weight of family members

Families are assigned consumption weights, defined by Swedish government agencies, to facilitate a comparison of the living standards of families with different demographic compositions. In 2004, the consumption weight was revised so that a second or third child between 0 and 19 years contributes less (0.42) consumption weight than the first child in a family (0.52). One adult in a family is assigned the value 1.00. If that adult is married/cohabiting, the second adult is assigned the value 0.51. All other adults (including children aged ≥ 20 years) are assigned 0.60. The age of the child does otherwise not influence the consumption weight in the revised classification scheme.

## References

[1] Neovius M, Arkema EV, Blomqvist P, Ekbom A, Smedby KE (2013). Patients with ulcerative colitis miss more days of work than the general population, even following colectomy. Gastroenterology.

[2] Di Thiene D, Rahman S, Helgesson M (2018). Healthcare use among immigrants and natives in Sweden on disability pension, before and after changes of regulations. Eur J Public Health.

[3] Ervasti J, Virtanen M, Lallukka T (2018). Trends in diagnosis-specific work disability before and after ischaemic heart disease: a nationwide population-based cohort study in Sweden. BMJ Open.

[4] Gyllensten H, Wiberg M, Alexanderson K (2018). Costs of illness of multiple sclerosis in Sweden: a population-based register study of people of working age. Eur J Health Econ.

[5] Rahman S, Wiberg M, Alexanderson K, Jokinen J, Tanskanen A, Mittendorfer-Rutz E (2018). Trajectories of antidepressant medication use in individuals before and after being granted disability pension due to common mental disorders—a nationwide register-based study. BMC Psychiatry.

[6] Farrants K, Kjeldgard L, Marklund S, Head J, Alexanderson K (2018). Sick leave before and after the age of 65 years among those in paid work in Sweden in 2000 or 2005: a register-based cohort study. J Int Med Res.

[7] Lallukka T, Ervasti J, Lundstrom E (2018). Trends in diagnosis-specific work disability before and after stroke: a longitudinal population-based study in Sweden. J Am Heart Assoc.

[8] Kavaliunas A, Danylaite Karrenbauer V, Gyllensten H (2017). Cognitive function is a major determinant of income among multiple sclerosis patients in Sweden acting independently from physical disability. Mult Scler J.

[9] Ervasti J, Virtanen M, Lallukka T (2017). Permanent work disability before and after ischaemic heart disease or stroke event: a nationwide population-based cohort study in Sweden. BMJ Open.

[10] Rod NH, Kjeldgard L, Akerstedt T (2017). Sleep apnea, disability pensions, and cause-specific mortality: a Swedish nationwide register linkage study. Am J Epidemiol.

[11] Kvillemo P, Mittendorfer-Rutz E, Branstrom R, Nilsson K, Alexanderson K (2017). Sickness absence and disability pension after breast cancer diagnosis: a 5-year nationwide cohort study. J Clin Oncol.

[12] Wiberg M, Marklund S, Alexanderson K (2017). Transitions between compensated work disability, joblessness, and self-sufficiency: a cohort study 1997–2010 of those jobless in 1995. Popul Res Policy Rev.

[13] Lallukka T, Ervasti J, Mittendorfer-Rutz E (2016). The joint contribution of diabetes and work disability to premature death during working age: a population-based study in Sweden. Scand J Public Health.

[14] Narusyte J, Bjorkenstam E, Alexanderson K, Ropponen A, Kjeldgard L, Svedberg P (2016). Occurrence of sickness absence and disability pension in relation to childbirth: a 16-year follow-up study of 6323 Swedish twins. Scand J Public Health.

[15] Narusyte J, Ropponen A, Alexanderson K, Svedberg P (2016). Genetic and environmental influences on disability pension due to mental diagnoses: limited importance of major depression, generalized anxiety, and chronic fatigue. Twin Res Hum Genet.

[16] Dorner TE, Alexanderson K, Svedberg P, Tinghog P, Ropponen A, Mittendorfer-Rutz E (2016). Synergistic effect between back pain and common mental disorders and the risk of future disability pension: a nationwide study from Sweden. Psychol Med.

[17] Wang M, Bjorkenstam C, Alexanderson K, Runeson B, Tinghog P, Mittendorfer-Rutz E (2015). Trajectories of work-related functional impairment prior to suicide. PLoS ONE.

[18] Dorner TE, Alexanderson K, Svedberg P, Ropponen A, Stein KV, Mittendorfer-Rutz E (2015). Sickness absence due to back pain or depressive episode and the risk of all-cause and diagnosis-specific disability pension: a Swedish cohort study of 4,823,069 individuals. Eur J Pain.

[19] Bjorkenstam E, Alexanderson K, Narusyte J, Kjeldgard L, Ropponen A, Svedberg P (2015). Childbirth, hospitalisation and sickness absence: a study of female twins. BMJ Open.

[20] Bjorkenstam E, Weitoft GR, Lindholm C, Bjorkenstam C, Alexanderson K, Mittendorfer-Rutz E (2014). Associations between number of sick-leave days and future all-cause and cause-specific mortality: a population-based cohort study. BMC Public Health.

[21] Bjorkenstam E, Narusyte J, Alexanderson K, Ropponen A, Kjeldgard L, Svedberg P (2014). Associations between childbirth, hospitalization and disability pension: a cohort study of female twins. PLoS ONE.

[22] Ropponen A, Alexanderson K, Svedberg P (2014). Part-time work or social benefits as predictors for disability pension: a prospective study of Swedish twins. Int J Behav Med.

[23] Ropponen A, Samuelsson A, Alexanderson K, Svedberg P (2013). Register-based data of psychosocial working conditions and occupational groups as predictors of disability pension due to musculoskeletal diagnoses: a prospective cohort study of 24,543 Swedish twins. BMC Musculoskelet Disord.

[24] Samuelsson A, Ropponen A, Alexanderson K, Svedberg P (2013). Psychosocial working conditions, occupational groups, and risk of disability pension due to mental diagnoses: a cohort study of 43,000 Swedish twins. Scand J Work Environ Health.

[25] Samuelsson A, Alexanderson K, Ropponen A, Lichtenstein P, Svedberg P (2012). Incidence of disability pension and associations with socio-demographic factors in a Swedish twin cohort. Soc Psychiatry Psychiatr Epidemiol.

[26] Wikman A, Wiberg M, Marklund S, Alexanderson K (2012). Activities and sources of income after a period of long-term sick leave—a population-based prospective cohort study. BMC Public Health.

[27] Naimi-Akbar A, Svedberg P, Alexanderson K, Ekstrand J, Sandborgh-Englund G (2012). Reliance on social security benefits by Swedish patients with ill-health attributed to dental fillings: a register-based cohort study. BMC Public Health.

[28] Floderus B, Hagman M, Aronsson G, Gustafsson K, Marklund S, Wikman A (2012). Disability pension among young women in Sweden, with special emphasis on family structure: a dynamic cohort study. BMJ Open.

[29] Samuelsson A, Ropponen A, Alexanderson K, Lichtenstein P, Svedberg P (2012). Disability pension among Swedish twins—prevalence over 16 years and associations with sociodemographic factors in 1992. J Occup Environ Med.

[30] Gustafsson K, Lundh G, Svedberg P, Linder J, Alexanderson K, Marklund S (2011). Disability, sickness, and unemployment benefits among long-term sickness absentees five years before, during, and after a multidisciplinary medical assessment. J Multidiscip Healthc.

[31] Brus O, Nordanskog P, Bave U (2017). Subjective memory immediately following electroconvulsive therapy. J ECT.

[32] Popiolek K, Brus O, Elvin T (2018). Rehospitalization and suicide following electroconvulsive therapy for bipolar depression-A population-based register study. J Affect Disord.

[33] Statistics Sweden (Swedish: Statistiska Centralbyrån LISA—Longitudinell integrationsdatabas för sjukförsäkrings- och arbetsmarknadsstudier. 2016. https://www.scb.se/LISA/. Accessed 25 March 2019.

[34] Ludvigsson JF, Almqvist C, Bonamy AE (2016). Registers of the Swedish total population and their use in medical research. Eur J Epidemiol.

[35] Ludvigsson JF, Otterblad-Olausson P, Pettersson BU, Ekbom A (2009). The Swedish personal identity number: possibilities and pitfalls in healthcare and medical research. Eur J Epidemiol.

[36] Ekbom A (2011). The Swedish multi-generation register. Methods Mol Biol.

[37] Befolkningens utbildning (Utbildningsregistret, UREG). English: education of the total population. Örebro2013.

[38] Barlow L, Westergren K, Holmberg L, Talback M (2009). The completeness of the Swedish cancer register: a sample survey for year 1998. Acta Oncol.

[39] Lidwall U, Marklund S (2011). Trends in long-term sickness absence in Sweden 1992–2008: the role of economic conditions, legislation, demography, work environment and alcohol consumption. Int J Soc Welf.

[40] Brus O, Cao Y, Gustafsson E (2017). Self-assessed remission rates after electroconvulsive therapy of depressive disorders. Eur Psychiatry.

[41] Rundgren S, Brus O, Bave U (2018). Improvement of postpartum depression and psychosis after electroconvulsive therapy: a population-based study with a matched comparison group. J Affect Disord.

[42] Bruze G, Holmin TE, Peltonen M (2018). Associations of bariatric surgery with changes in interpersonal relationship status: results from 2 Swedish cohort studies. JAMA Surg.

[43] Chen L, Eloranta S, Martling A (2018). Short- and long-term risks of cardiovascular disease following radiotherapy in rectal cancer in four randomized controlled trials and a population-based register. Radiother Oncol.

[44] Ng WL, Peeters A, Naslund I (2017). Change in use of sleep medications after gastric bypass surgery or intensive lifestyle treatment in adults with obesity. Obesity (Silver Spring).

[45] Bruze G, Ottosson J, Neovius M, Naslund I, Marsk R (2017). Hospital admission after gastric bypass: a nationwide cohort study with up to 6 years follow-up. Surg Obes Relat Dis.

[46] Khalili H, Neovius M, Ekbom A (2016). Oral contraceptive use and risk of ulcerative colitis progression: a nationwide study. Am J Gastroenterol.

[47] Hagman E, Danielsson P, Brandt L, Svensson V, Ekbom A, Marcus C (2017). Childhood obesity, obesity treatment outcome, and achieved education: a prospective cohort study. J Adolesc Health.

[48] Ludvigsson JF, Welander A, Lassila R, Ekbom A, Montgomery SM (2007). Risk of thromboembolism in 14,000 individuals with coeliac disease. Br J Haematol.

[49] Zugna D, Richiardi L, Akre O, Stephansson O, Ludvigsson JF (2010). A nationwide population-based study to determine whether coeliac disease is associated with infertility. Gut.

[50] Neovius M, Bruze G, Jacobson P (2018). Risk of suicide and non-fatal self-harm after bariatric surgery: results from two matched cohort studies. Lancet Diabetes Endocrinol.

[51] Sundstrom J, Bruze G, Ottosson J, Marcus C, Naslund I, Neovius M (2017). Weight loss and heart failure: a nationwide study of gastric bypass surgery versus intensive lifestyle treatment. Circulation.

[52] Eriksson JK, Wallman JK, Miller H (2016). Infliximab versus conventional combination treatment and seven-year work loss in early rheumatoid arthritis: results of a randomized Swedish trial. Arthritis Care Res (Hoboken).

[53] Larsson H, Sariaslan A, Langstrom N, D’Onofrio B, Lichtenstein P (2014). Family income in early childhood and subsequent attention deficit/hyperactivity disorder: a quasi-experimental study. J Child Psychol Psychiatry.

[54] Sariaslan A, Larsson H, D’Onofrio B, Langstrom N, Lichtenstein P (2014). Childhood family income, adolescent violent criminality and substance misuse: quasi-experimental total population study. Br J Psychiatry.

[55] Olofsson T, Petersson IF, Eriksson JK (2017). Predictors of work disability after start of anti-TNF therapy in a national cohort of Swedish patients with rheumatoid arthritis: Does early anti-TNF therapy bring patients back to work?. Ann Rheum Dis.

[56] Chen L, Glimelius I, Neovius M (2015). Risk of disability pension in patients following rectal cancer treatment and surgery. Br J Surg.

[57] Glimelius I, Ekberg S, Linderoth J (2015). Sick leave and disability pension in Hodgkin lymphoma survivors by stage, treatment, and follow-up time—a population-based comparative study. J Cancer Surv.

[58] Neovius M, Simard JF, Klareskog L, Askling J (2011). Sick leave and disability pension before and after initiation of antirheumatic therapies in clinical practice. Ann Rheum Dis.

[59] Gyhagen M, Al-Mukhtar Othman J, Akervall S, Nilsson I, Milsom I (2018). The symptom of vaginal bulging in nulliparous women aged 25–64 years: a national cohort study. Int Urogynecol J.

[60] Al-Mukhtar Othman J, Akervall S, Milsom I, Gyhagen M (2017). Urinary incontinence in nulliparous women aged 25–64 years: a national survey. Am J Obstet Gynecol.

[61] Beckman K, Mittendorfer-Rutz E, Lichtenstein P (2016). Mental illness and suicide after self-harm among young adults: long-term follow-up of self-harm patients, admitted to hospital care, in a national cohort. Psychol Med.

[62] Beckman K, Mittendorfer-Rutz E, Waern M, Larsson H, Runeson B, Dahlin M (2018). Method of self-harm in adolescents and young adults and risk of subsequent suicide. J Child Psychol Psychiatry.

[63] Sariaslan A, Langstrom N, D’Onofrio B, Hallqvist J, Franck J, Lichtenstein P (2013). The impact of neighbourhood deprivation on adolescent violent criminality and substance misuse: a longitudinal, quasi-experimental study of the total Swedish population. Int J Epidemiol.

[64] Butwicka A, Sariaslan A, Larsson H (2018). No association between urbanisation, neighbourhood deprivation and IBD: a population-based study of 4 million individuals. Gut.

[65] Sariaslan A, Larsson H, D’Onofrio B, Langstrom N, Fazel S, Lichtenstein P (2015). Does population density and neighborhood deprivation predict schizophrenia? A nationwide Swedish family-based study of 2.4 million individuals. Schizophr Bull.

[66] Sariaslan A, Fazel S, D’Onofrio BM (2016). Schizophrenia and subsequent neighborhood deprivation: revisiting the social drift hypothesis using population, twin and molecular genetic data. Transl Psychiatry.

[67] Murley C, Mogard O, Wiberg M (2018). Trajectories of disposable income among people of working ages diagnosed with multiple sclerosis: a nationwide register-based cohort study in Sweden 7 years before to 4 years after diagnosis with a population-based reference group. BMJ Open.

[68] Mayer-Davis EJ, Lawrence JM, Dabelea D (2017). Incidence trends of type 1 and type 2 diabetes among youths, 2002–2012. N Engl J Med.

[69] Persson S, Dahlquist G, Gerdtham UG, Steen Carlsson K, Swedish Childhood Diabetes Study G (2018). Why childhood-onset type 1 diabetes impacts labour market outcomes: a mediation analysis. Diabetologia.

[70] Loven I, Steen Carlsson K (2017). Early onset of type 1 diabetes and educational field at upper secondary and university level: Is own experience an asset for a health care career?. Int J Environ Res Public Health.

[71] Persson S, Gerdtham UG, Steen Carlsson K, Swedish Childhood Diabetes Study G (2016). Labor market consequences of childhood onset type 1 diabetes. Econ Hum Biol.

[72] Persson S, Dahlquist G, Gerdtham UG, Steen Carlsson K (2013). Impact of childhood-onset type 1 diabetes on schooling: a population-based register study. Diabetologia.

[73] Steen Carlsson K, Landin-Olsson M, Nystrom L (2010). Long-term detrimental consequences of the onset of type 1 diabetes on annual earnings—evidence from annual registry data in 1990–2005. Diabetologia.

[74] Eriksson JK, Neovius M, Bratt J (2013). Biological vs. conventional combination treatment and work loss in early rheumatoid arthritis: a randomized trial. JAMA Intern Med.

